# Three-Dimensional Computer-Aided Detection of Microcalcification Clusters in Digital Breast Tomosynthesis

**DOI:** 10.1155/2016/8651573

**Published:** 2016-05-04

**Authors:** Ji-wook Jeong, Seung-Hoon Chae, Eun Young Chae, Hak Hee Kim, Young-Wook Choi, Sooyeul Lee

**Affiliations:** ^1^SW·Content Research Laboratory, Electronics & Telecommunications Research Institute, 218 Gajeongno, Yuseong-gu, 34129 Daejeon, Republic of Korea; ^2^Department of Radiology and Research Institute of Radiology, Asan Medical Center, University of Ulsan College of Medicine, 88 Olympic-ro 43-gil, Songpa-gu, 05505 Seoul, Republic of Korea; ^3^Advanced Medical Device Research Division, Korea Electrotechnology Research Institute, 111 Hanggaul-ro, Sangnok-gu, Ansan-si, 15588 Gyeonggi-do, Republic of Korea

## Abstract

We propose computer-aided detection (CADe) algorithm for microcalcification (MC) clusters in reconstructed digital breast tomosynthesis (DBT) images. The algorithm consists of prescreening, MC detection, clustering, and false-positive (FP) reduction steps. The DBT images containing the MC-like objects were enhanced by a multiscale Hessian-based three-dimensional (3D) objectness response function and a connected-component segmentation method was applied to extract the cluster seed objects as potential clustering centers of MCs. Secondly, a signal-to-noise ratio (SNR) enhanced image was also generated to detect the individual MC candidates and prescreen the MC-like objects. Each cluster seed candidate was prescreened by counting neighboring individual MC candidates nearby the cluster seed object according to several microcalcification clustering criteria. As a second step, we introduced bounding boxes for the accepted seed candidate, clustered all the overlapping cubes, and examined. After the FP reduction step, the average number of FPs per case was estimated to be 2.47 per DBT volume with a sensitivity of 83.3%.

## 1. Introduction

Recently, there have been several reports that the acquisition of several projection views (PVs) of the compressed breast using a conventional full-field digital mammography (FFDM) detector is sufficient to reconstruct the total DBT images at a total radiation dose comparable to that used in mammography [1, 2]; however, the reconstructed 3D DBT volume contains artifacts from missing information, regardless of the reconstruction technique applied [3–5]. Despite these shortcomings, it is expected that DBT can reduce the overlapping breast tissue effect, which is usually considered to be a limiting factor for lesion detection and characterization in FFDM [1, 6, 7].

There has been a controversy as to whether or not the cancer detection performance through prospective clinical trials has found an increase in sensitivity with a moderate increase in the call-back rate [8–10]. As a consistent second reader, CADe may be helpful by detecting lesions in DBT missed by radiologists owing to the large volume of the image data, as well as a number of other factors [11]. The detection of MCCs in DBT volumes by radiologists may be more difficult compared with mammography for two reasons: the number of MCs on each reconstructed slice will be fewer than the total number of MC clusters, making it less apparent. CADe may be of particular interest in DBT for MC detection by adopting a maximum intensity projection (MIP) method. MCs may appear blurred from many factors including an inaccurate system geometry, focal spot motion, and patient motion. There have been several feasibility studies recently regarding CADe for MC detection in DBT [12, 13].

It is also important to reduce the number of false negatives during the search for MCCs in a 3D DBT volume, which may be more demanding than for mammograms. For these reasons, CADe may play an even more important role in MC detection in DBT than in mammography by automatically searching for MCCs in a 3D DBT image volume within a relatively short period of time. There have been a number of studies regarding the development of CADe techniques for the detection of masses in DBT [11, 14–17]. Compared with the detection of masses, preliminary researches regarding the detection of MCCs on DBT have been reported [12, 18–23].

Reiser and coworkers back-projected binarized PVs containing detected MCs into a 3D volume to conduct an MIP transformation for second-stage detection [18]. Features were extracted and a false-positive (FP) reduction step was conducted with a sensitivity of 86% with 1.3 FP clusters per DBT volume. Bernard and others developed a detection algorithm of MCCs on filtered back-projection reconstructed slices [21] enhanced by convolving the image volume with a Mexican hat wavelet with a sensitivity of 85% at an average of 1.4 FP marks per breast volume. Park and coworkers detected MCCs on both individual PVs with a sensitivity of 70% at an average of 3.99 FPs per volume and individual reconstructed slices with a sensitivity of 86% at an average of 15.9 FPs per volume [19]. Sahiner and coworkers investigated the detection of MCCs in the reconstructed DBT volume using an enhanced-modulated 3D multiscale calcification response function and SNR enhancement [13].

In this paper, a simple and efficient FP reduction scheme coupled with a detection algorithm for MCCs in a reconstructed DBT volume using 3D objectness- and SNR-enhanced images is suggested [13, 23]. For a dataset of two-view DBTs of 69 breasts with or without MCCs, a view-based sensitivity of 83.3% was achieved at 2.47 FPs per DBT volume.

## 2. Data Acquisition

The patient recruitment protocol was approved by IRB. Breast imaging patients of the breast imaging research laboratory at Asan Medical Center (Seoul, Korea) who were recommended for breast biopsy based on suspicious mammographic breast masses and microcalcifications were eligible. Written informed consent was obtained from each patient. We acquired the DBT scans of 15 PV images over a ±21 angular range in 2.8 increments through a step-and-shoot operation using the prototype DBT system for breast imaging research fabricated by KERI (Ansan, Korea) [25]. The DBT system has a flat panel digital detector with dimensions of 14.40 cm × 25.92 cm and a pixel pitch of 0.0748 mm × 0.0748 mm [26–28]. The 3D DBT volumes were reconstructed at a 1 mm slice interval with a pixel pitch of 0.1 mm × 0.1 mm using the FDK filtered back-projection reconstruction technique [29]. DBT scans of the 69 breasts were acquired in both craniocaudal and mediolateral oblique views prior to a biopsy and the location of the biopsy-proven MCC was marked by an experienced radiologist using clinical mammograms and the biopsy report as references. A total of 19 MCCs were identified on the 138 DBT scans.

## 3. Prescreening Step

The MCs are first enhanced using a multiscale Hessian enhancement with an object-type response function and an SNR enhancement with combination of several simple digital filters in the reconstructed DBT volume (Im⁡(*x*, *y*, *z*)) [13]. Then, the resulting Hessian-based object-type response volume is again voxel-wise convolved with the SNR-enhanced volume, and a connected-component segmentation process [30] is performed to extract the clustering seed objects for the MCCs, which are described in Section 5. Before the prescreening step, we subsampled the DBT images in the *x*-*y* direction by 10 and the maximum grey level value was chosen as a representative value to reduce the computational load; its effect on the detection accuracy is discussed later in this paper.

### 3.1. Multiscale Object-Type Response

There is an observation that all three eigenvalues of the Hessian matrix [30, 31] near the center of a spherically symmetric lesion with positive contrast are given negative and are nearly equal to each other in the case of spherical shape, whereas the Hessian matrix for voxels that are a part of other types of shapes, such as lines or planes, will give unequal eigenvalues. In practice, to optimize the Hessian enhancement process for objects at different scales and reduce the computational noise from the estimation of second-order derivatives in the Hessian operator calculations, the image *I*(*x*, *y*, *z*) is first convolved with a 3D Gaussian smoothing filter giving a smoothed image *G*(*x*, *y*, *z*) and Hessian matrix *H*_*εδ*_ setup as follows:

(1) $$\begin{array}{c} H_{\varepsilon \delta }=\frac{\partial^{2}G\left(x,y,z\right)}{\partial \varepsilon \partial \delta }, \end{array}$$

where (*ε*, *δ*) is chosen among the Cartesian axes of *x*, *y*, and *z*, alternatively. Then, the Hessian matrix (*H*_*αβ*_; *α*, *β* ∈ {*x*, *y*, *z*}) is diagonalized and the eigenvalues {(*λ*_1_, *λ*_2_, *λ*_3_)∣0 ≥ *λ*_3_ ≥ *λ*_2_ ≥ *λ*_1_} are specified in ascending order.

To enhance a spherically symmetric object, a response *O*({*λ*_*i*_}; *σ*) at a Gaussian scale *σ* is defined using

(2) Oλi;σ=1−exp⁡−λ122α2λ2λ3·1−exp⁡−λ12+λ22+λ322γ2,

where the selected values for the objectness parameters of *α* and *γ* are 0.1 and 3.0, respectively [32]. We find that the selection of typically chosen functional type for an object response is enough to give sufficiently accurate detection results comparable to other researches.

**Figure 1:**
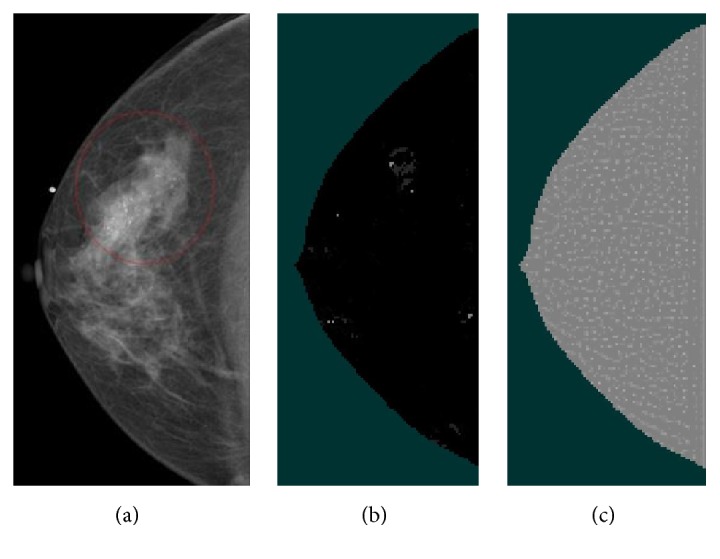
(a) A mammogram containing MCCs. The ROI drawn by a radiologist is shown in the red circle. (b) A multiscale Hessian-enhanced image. (c) An SNR-enhanced image.

Then, a response vector *R* = {*O*(*λ*_*σ*1_), *O*(*λ*_*σ*2_),…, *O*(*λ*_*σN*_)} at multiple scales {*σ*} = {*σ*1, *σ*2,…, *σN*} is obtained at every (*x*, *y*, *z*). In this study, we chose *N* = 3 to reduce the computational load. After the optimal index for scale *i* ^*∗*^ = arg max_*i*_{*O*(*λ*_*σi*_)} was estimated, the multiscale object-type response (MOR) at every voxel of (*x*, *y*, *z*) was then evaluated as *O*(*x*, *y*, *z*) = *O*(*λ*_*σi*_^*∗*^).  Figure 1(b) illustrates the filtering process to enhance the objectness using the multiscale Hessian matrix.

### 3.2. 3D SNR Enhancement

**Figure 2:**
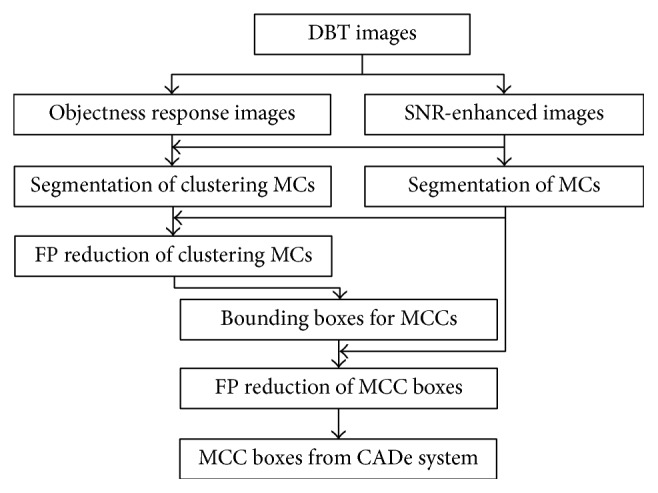
The block diagram of our CADe system.

From the clinical observations, many visible MCs are closely related with grey level fluctuations, and we introduced an SNR enhancement preprocessing step to each two-dimensional DBT slice independently. It consists of a combination of three linear mean filters centered at the calcification candidate, *F*_1_, *F*_2_, and *F*_3_, of sizes *M*_1_ × *M*_1_, *M*_2_ × *M*_2_, and *M*_3_ × *M*_3_, respectively, where *M*_1_ = 15, *M*_2_ = 7, and *M*_3_ = 3, to define a single band-pass filter before convolution with the image and extracting the signal intensity relative to the slowly varying background image intensity [13]. In order to remove an artifact resulting from the inclusion of pixels right near the candidate pixel, *F*_2_ is subtracted from *F*_1_ filter. Then, the combined band-pass filter was convolved with DBT volume as illustrated in Figure 1(c). Overall block diagram for our CADe system of MCCs in DBT images is given in Figure 2.

## 4. MCC Detection

In the MCC detection step, we firstly weighted *O*(*x*, *y*, *z*) with SNR-enhanced voxel value of SNR(*x*, *y*, *z*), where SNR(*x*, *y*, *z*) is given by SNR(*x*, *y*, *z*) = *I*(*x*, *y*, *z*) ⊗ *F*(*x*, *y*), and *F*(*x*, *y*) is a resulting 2D digital filter in order to obtain the multiscale objectness response (MOR). Since both the multiscale calcification response and the SNR enhancement are intended to highlight the MCs, it may be expected that their product will improve the microcalcification detection.

**Figure 3:**
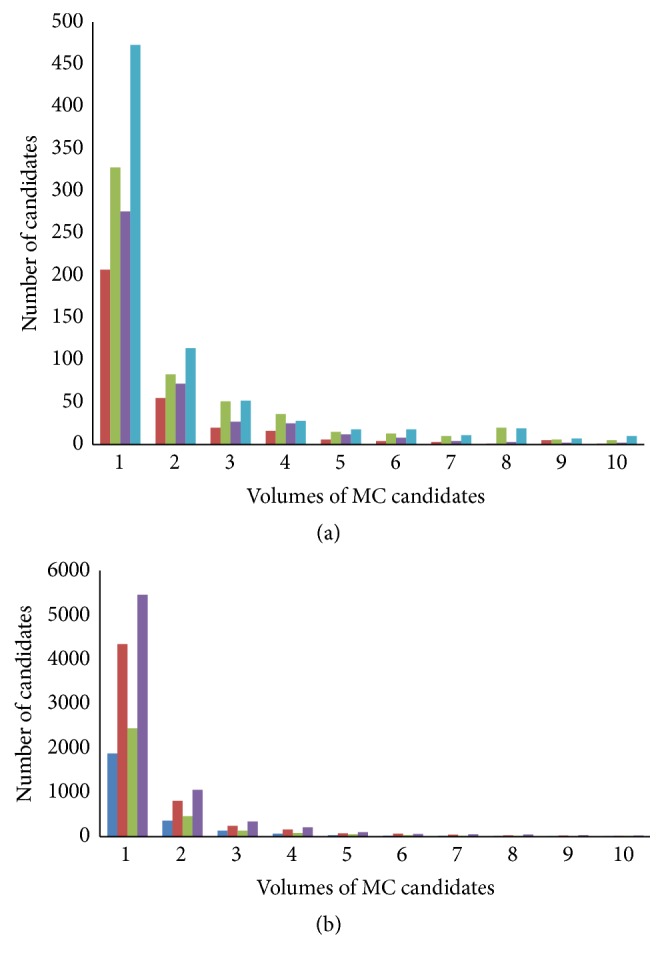
(a) Volume distributions of segmented candidates from (a) MOR image and (b) SNR image. Blue, brown, green, and violet colored bars are for RMLO, LMLO, RCC, and LCC modalities of a DBT image, respectively.

Over MOR(*x*, *y*, *z*), a connected-component segmentation technique was performed to detect about 500 connected objects as the initial seed objects [30, 33]. Voxels that were above the binarizing threshold were marked and grouped into 3D connected objects to give a labeled image. The labeled image was again converted to the labeled map to examine the shape attributes of each segmented and labeled object. The initial threshold was chosen to be relatively high enough to detect only about 500 connected objects, which are defined as cluster seed objects below as exemplified in Figure 3.

The noise around each individual MC candidate was estimated using the SNR3D(*x*, *y*, *z*) images as follows. The squared noise level *σ* ^2^(*x*, *y*, *z*) at each input voxel of SNR(*x*, *y*, *z*) was evaluated from the standard deviation of the grey level value distribution in the neighborhood of that pixel. The SNR of the MC candidate was then calculated as the ratio of *σ* ^2^(*x*, *y*, *z*) and the local mean value *m*_loc_(*x*, *y*, *z*) at the same voxel as follows:

(3) $$\begin{array}{c} SNR3D\left(\;x,y,z\;\right)=\frac{\sigma^{2}{\left(x,y,z\right)}_{SNR\left(x,y,z\right)}}{m_{loc}{\left(x,y,z\right)}_{SNR\left(x,y,z\right)}}, \end{array}$$

where the subscripts indicate the corresponding local operator performed on the given 3D image. The individual MC candidates were also labeled using a connected-component segmentation algorithm and an SNR threshold value of 3.2 was chosen for the binarization of the SNR3D(*x*, *y*, *z*) image to locate about 5,000 MC candidates, independent of the cluster seed object detection process.

**Figure 4:**
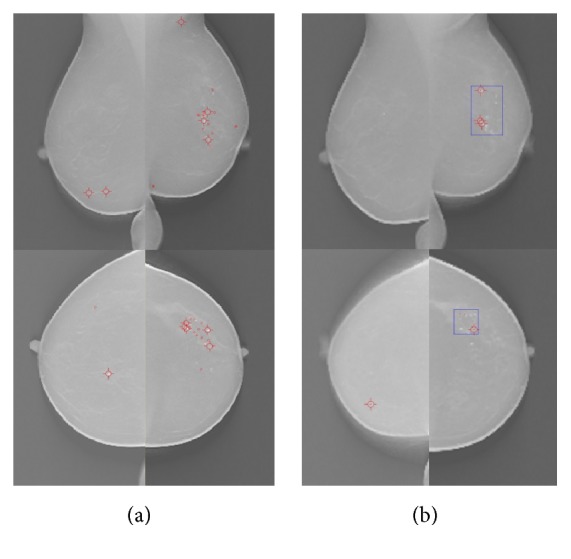
Snapshots of our MCC detection system (a) before and (b) after clustering step. Red circles are for the clustering MC candidates extracted from an MOR image after the prescreening step based on the analysis of the individual MC candidates extracted from an SNR image. Blue square means a bounding box after the prescreening step. Left top, right top, left bottom, and right bottom images are for RLMO, LMLO, RCC, and LCC modalities of a DBT image.

Then, an MC clustering process was applied to choose the MCC candidates as follows. Note that only the clustering seed objects segmented from the MOR images were considered as the clustering center. Starting with each cluster seed object, individual MC candidates satisfying the clustering criteria of their distances being within 5 mm from the cluster center were included as cluster members.  Figure 4 shows a snapshot of our MCC detection system before MC clustering and FP reduction step.

## 5. False-Positive Reduction

As a first step to reduce the FPs for the MCC candidates, we applied a rule-based classifier with two rules related to the voxel sum of the individual MCs and the number of cluster seed candidates in the neighborhood of the candidate cluster. The voxels of individual MC candidates within a 5 mm radius of the cluster seed candidate were counted and the first rule specifies that if this number is less than 9, the cluster seed candidate will be eliminated. We also counted the number of nearby cluster seed candidates within a 5 mm radius of the cluster candidate under consideration. The second rule specifies that if this number is less than 2, the cluster candidate will be eliminated.

Second, the cubes minimally containing the MCs were generated and clustered for a further FP reduction. To qualify the clustered MCs, a bounding cube was generated for each accepted seed candidate. The overlapping cubes were combined and examined to determine whether the number of combined cubes is larger than one. Next, we examined the number and voxel sum of the included individual MC candidates contained in each cube. When the number of the included individual MC candidates within the resulting MCC cube was less than 80 or the total volume of the MC candidates was less than 140 mm^3^, the combined cube was eliminated.

In this study, a suggested MCC candidate on the DBT image was considered as true positive, if the overlapped volume between the annotated gold standard and the detected MCC candidate is larger than zero, to simplify the volumetric analysis between them. Further improvement of the FP reduction algorithm using the elaborated volumetric analysis as a next step will be reported in the near future.

## 6. Results and Discussion

**Figure 5:**
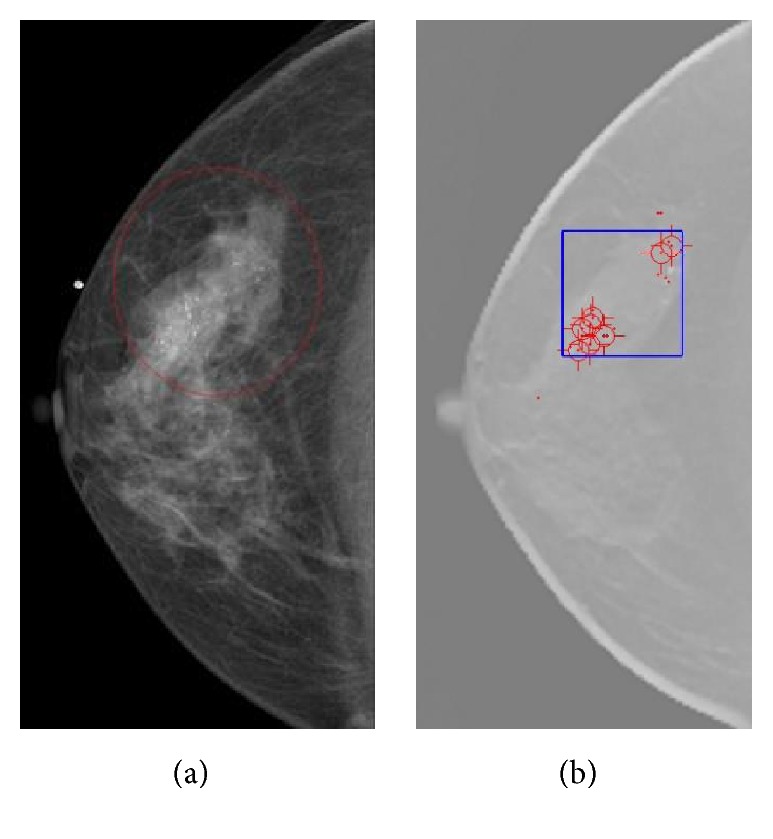
(a) A mammogram containing MCCs. The ROI drawn by a radiologist is shown in the red circle. (b) A DBT slice with the suggested MCC bounding cube. The blue square containing the MCCs was automatically determined during the CADe calculation. Red dots or circles indicate the MC seed objects before the FP reduction step.

As shown in Figure 4, our detection and screening system for the MCCs in DBT successfully suggests the outlining range of inspection. Using view-based scoring, the average number of FPs using the datasets was estimated to be 2.47 per DBT volume at an 83.3% sensitivity, which was found to be comparable with other pioneering researches [12, 13]. In particular, it is notable that the FPs for the DBT volumes with MCCs proven to be positive were found to be 2.24, lower than 2.56 for the DBT volumes without an MCC [34]. It is noted that our CADe system gives less FPs with the true-positive lesion marking than with normal breast images, indicating that our algorithm should be improved further to give the lower specificity or less FPs with the same sensitivity results. It is well known that the higher rate of FPs of the breast cancer diagnosis usually leads to increased psychological and economical burden, meaning that the patients undergo unnecessary and exhaustive diagnostic procedures. It is also notable that the clinical criterion of FPs per case for CADe based on any commercial medical imaging modality is eventually under or about to be one-per-case, and the mammography CADe of the breast cancer has been proven to satisfy such a criterion. Our results compared with the clinical gold standards marked by a radiologist (as exemplified in Figure 5) indicate that the feasibility of automated detection algorithm of MCCs in reconstructed DBT volumes coupled with the relatively simplified clustering and FP reduction algorithm, however, is also necessary to fine-tune the prescreening parameters to reduce the number of FPs.

**Figure 6:**
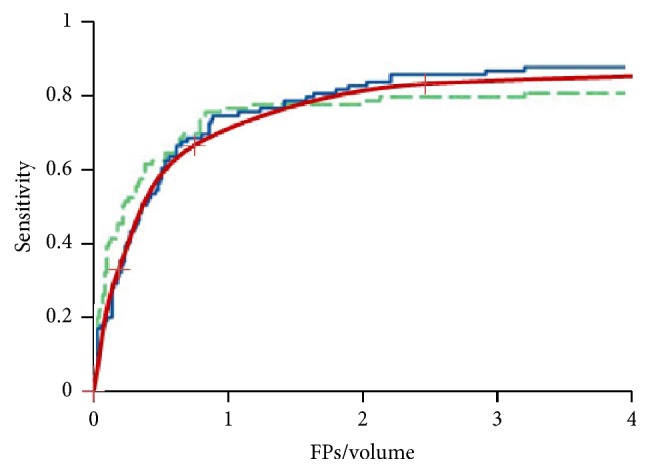
The overall performance of the MCC detection and FP reduction algorithm in terms of the FROC curve. Solid brown line is for the ROC using our CADe algorithm. Solid blue and dashed green lines are for the ROCs using the FP reduction algorithm involving convolution neural network features with the DBT volume and the digital mammography [24].

In principle, our approach basically utilizes the combination of two microcalcification enhancement processes: (1) 3D objectness enhancement of the MC response based on a multiscale Hessian analysis and (2) SNR enhancement based on a combination of linear boxcar filters. The FPs per DBT volume were counted from the dataset after two FP reduction steps, and the free response receiver operating characteristics (FROC) curve for the detection system is shown in Figure 6. The area under the FROC curve normalized to 20.44 FPs per DBT volume at a sensitivity of 100.0% was estimated to be 0.88 [35–38], implying that our results are in quite reasonable agreement with previous researches in spite of several assumptions and the setting of the evaluation parameters [39–41], however, giving slow convergence of the sensitivity as FPs increase.

Our CADe system contains a large number of parameters for prescreening, clustering, and FP reduction stages. In this preliminary study, we focused on the effect of the FP reduction threshold parameters. The binarizing threshold values for the MOR volume and the SNR-enhanced volume were chosen empirically, implying that further studies with a larger DBT image set to optimize the control parameters may improve the MC detection performance [42–44].

The MOR function used in this study was introduced for spherically symmetric objects; however, pathology-proven MCs will have a variety of shapes, including elongated, stellated, and irregular shapes. In addition, the interplanar artifacts resulting from the adopted limited-angle reconstruction algorithm to obtain DBT image may distort the shape of the MCs in the depth direction and influence the performance of the applied segmentation method probably giving lower sensitivity of MCC detection [22]. Further studies regarding the anisotropic properties of the SNR distribution in the depth direction are also desired.

It should be commented that there have been some notable reports regarding MC detection on PVs [18, 19]. The MC detection on each two-dimensional PV is supposed to be independent of the specific reconstruction method, meaning that its detection results can be compared without the interplanar artifacts originating from the incomplete reconstruction algorithm for the DBT images. Tomosynthesis reconstruction with multiple noisy PVs can play a role in the prescreening step in the clinical MC detection approach by increasing the SNR of the targets.

It is notable that several pioneering reports involving various imaging modalities, such as three-dimensional DBT volume itself [24] and the planar projection view images [45], have been published up to now; however, the CADe for DBT images seems to be not outperforming that for digital mammography within near future. It is also worth commenting that, recently, there has been a report of a multimodal joint-CADe algorithm involving both DBT and projection view images [46].

Further research using the independently acquired DBT volume dataset is in progress to validate our proposed FP reduction algorithm.

## 7. Conclusion 

We developed a CADe system with a simplified FP reduction scheme for the detection of MCCs in reconstructed DBT volumes. The result of our proposed MCC detection algorithm is a promising approach, giving detection results comparable to other researches. Ongoing researches include further optimization of the FP reduction parameters using a large dataset and the 2D-3D hybridized detection on both PVs and the reconstructed volume. It is expected that, with further study on the CADe algorithm to improve the detection accuracy, the CADe system may play the role of a second reader by assisting radiologists in the detection of MCCs in DBT in the near future.
